# Effect of Composition and Impurities on the Phosphorescence of Green-Emitting Alkaline Earth Aluminate Phosphor

**DOI:** 10.1371/journal.pone.0145434

**Published:** 2016-01-05

**Authors:** Doory Kim, Han-Eol Kim, Chang-Hong Kim

**Affiliations:** 1 Department of Chemistry and Chemical Biology, Harvard University, Cambridge, Massachusetts, United States of America; 2 Gwangju Institute of Science and Technology, Gwangju, South Korea; 3 Korea Institute of Science and Technology, Seoul, South Korea; Aligarh Muslim University, INDIA

## Abstract

Recent improvements to SrAl_2_O_4_:Eu^2+^, Dy^3+^ phosphors have enabled the use of luminescent hosts with a stable crystal structure and high physical and chemical stability, thus overcoming the bottleneck in the applicability of ZnS:Cu phosphors. However, enhancement of afterglow lifetime and brightness in SrAl_2_O_4_:Eu^2+^, Dy^3+^ phosphors remains a challenging task. Here, we have improved the afterglow characteristics in terms of persistence time and brightness by a systematic investigation of the composition of Eu-doped alkaline earth aluminate SrAl_2_O_4_:Eu^2+^, Dy^3+^ crystals. We found that a Dy^3+^/Eu^2+^ ratio of ~2.4 and ~0.935 mol Eu^2+^ (per mol of SrAl_2_O_4_) gave the brightest and longest emissions (11% and 9% increase for each). Doping with Si^4+^ also resulted in a slight increase in brightness up to ~15%. Doping with alkali metal or alkaline earth metal significantly enhanced the phosphorescence intensity. In particular, doping with 0.005 mol Li^+^ (per mol of SrAl_2_O_4_) alone boosted the phosphorescence intensity to 239% of the initial value, as compared to that observed for the non-doped crystal, while doping with 0.01 mol Mg^2+^ and 0.005 mol Li^+^ (per 1 mol SrAl_2_O_4_) boosted the phosphorescence intensity up to 313% of the initial value. The results of this investigation are expected to act as a guideline for the synthesis of bright and long persistent phosphors, and facilitate the development of persistent phosphors with afterglow characteristics superior to those of conventional phosphors.

## Introduction

Phosphorescent materials have attracted considerable attention with respect to a wide range of applications in organic light emitting devices (OLEDs) and glow-in-the-dark materials, which are charged with bright light such as room light. Unlike a fluorescent material, a phosphorescent material releases generally weak light, very slowly in the dark, due to forbidden energy state transitions, instead of re-emitting the light immediately. Therefore, the development of phosphorescent emitters with a high phosphorescence quantum yield at room temperature has been considered important. ZnS:Cu phosphor is a well-known long phosphorescent phosphor, but it does not maintain its phosphorescence for more than a few hours, and is not bright and chemically stable enough for many applications. In order to overcome this limit, strontium aluminates have been developed[1–5]. The luminance of strontium aluminates is approximately 10 times greater than that of zinc sulfide [5,6], and they have intrinsically high chemical and physical stability as well as moisture resistance. Although many recent studies have improved the phosphorescence quantum yield of strontium aluminates by the use of activators and co-activators [7–12], it is challenging to develop long and enhanced afterglow phosphors. In this work, we have investigated the effects of various doping compositions and impurities on the phosphorescence of green-emitting alkaline earth aluminate phosphor (SrAl_2_O_4_:Eu^2+^,Dy^3+^) and improved its phosphorescence characteristics. The properties of phosphorescence emission are largely dominated by the effect of crystal-field symmetry on the excitation state of the activator Eu^2+^. Therefore, we compared various compositions of the activator (Eu^2+^) and co-activator (Dy^3+^), and impurities to find optimal conditions for improving the brightness and decay time of the green-emitting alkaline earth aluminate phosphor. Thus, we succeeded in developing a new phosphor, SrAl_2_O_4_:Eu^2+^,Dy^3+^, which shows extremely bright and long-lasting phosphorescence.

## Results

### Composition of activator and co-activator

**Fig 1:**
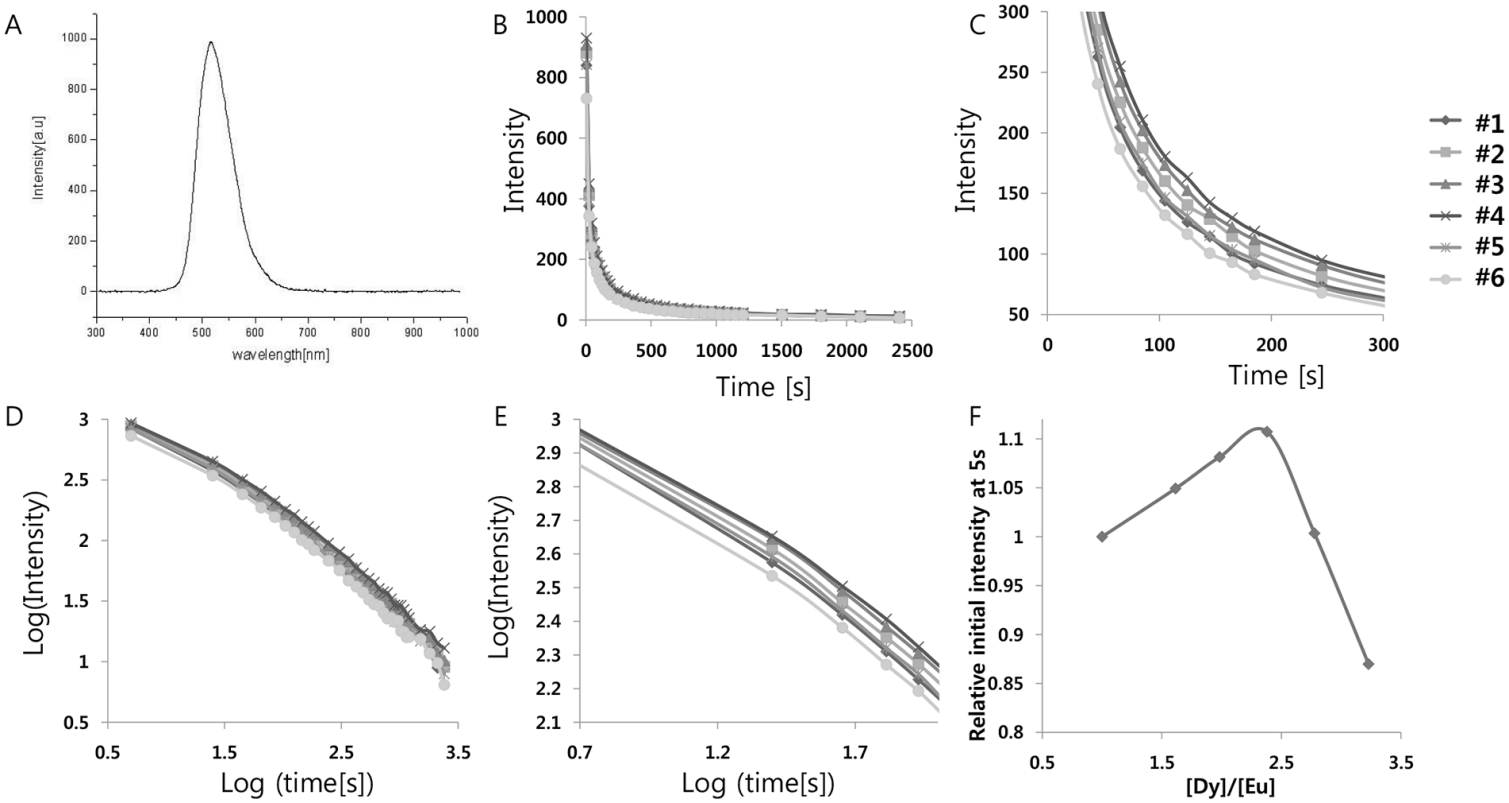
(A) Emission spectrum of strontium aluminate crystals. (B) Decay curves depending on [Dy^3+^]/[Eu^2+^]concentration. (C) Magnified views of the graph in (B). (D) Decay curves in log scale depending on [Dy^3+^]/[Eu^2+^]concentration. (E) Magnified views of the graph in (D). (F) Relative initial intensity measured at 5s (relative values where the value of control sample #1 is 1.0) depending on [Dy^3+^]/[Eu^2+^]concentration.

The SrAl_2_O_4_:Eu^2+^ system exhibits a broadband emission spectrum peaking at 520 nm, as shown in Fig 1, which is attributed to the 4f→ 5d transition of Eu^2+^. The incorporation of Dy^3+^ ion into the SrAl_2_O_4_:Eu^2+^ system as a co-activator is thought to produce very bright and long-lasting phosphorescence at room temperature because of the creation of highly dense hole trapping levels at the optimal depth. Because f–d transitions are very sensitive to crystal field distortion [13], the phosphorescence emission mechanism depends on the crystal-field symmetry upon the excitation states of the activator and co-activator. Therefore, chemical equilibrium between the activator and the co-activator may play an important role in changing the phosphorescence properties. As an initial optimization, we characterized the effect of activator and co-activator composition on the phosphorescence intensity. We measured the afterglow for various Dy^3+^/Eu^2+^ molar ratios ranging from 1 to 3, by changing only the Dy^3+^ concentration first (Table 1). The samples were irradiated with 365 nm light for 5 min and the decay curve of the afterglow at 520 nm, which corresponds to the peak of the 5d → 4f transition, was measured at room temperature (Fig 1B–1E). We further computed the lifetimes, which are the inverse of the decay rates, by fitting the decay curves with three exponential components having different decay times as previously reported [8]. These photophysical results are presented in Table 2. These different emission lifetimes are known to result from the different depths of the Dy^3+^ trap levels in the host structure [8,14]. Decay times do not vary greatly with varying compositions, but the initial afterglow intensity measured at 5 s changes significantly with the Dy^3+^/Eu^2+^ molar ratio of the starting materials. From these results, it is apparent that the Eu^2+^- and Dy^3+^-doped strontium aluminates with a Dy^3+^/Eu^2+^ ratio of ~2.4 shows the strongest persistent luminescence, when the ratio is varied from 1 to 3.2. This ratio is higher than the previously known value [8]. When the Dy^3+^/Eu^2+^ ratio is less than 2.4, the amount of Dy^3+^ contributing to afterglow characteristics, relative to the amount of Eu^2+^, may not be sufficient to obtain excellent initial afterglow characteristics. Therefore, when the concentration of Dy^3+^ ions relative to the Eu^2+^ ions increases, the initial afterglow intensity is enhanced, probably due to an increase in the concentration of hole traps with Dy^3+^ doping. However, when the Dy^3+^/Eu^2+^ ratio is greater than 2.4, the amount of Eu^2+^ contributing to phosphorescence characteristics becomes less than the amount of Dy^3+^ contributing to afterglow characteristics; the by-product DyAlO_3_ could be produced from the residual Dy^3+^ present in excess of the soluble limits, thus deteriorating the afterglow luminance characteristics [7].

**Table 1:** Various nominal activator(Eu^2+^) and co-activator(Dy^3+^) compositions of the strontium aluminate crystals.

SrAl_2_O_4_:Eu_a_,Dy_b_	#1	#2	#3	#4	#5	#6
a	0.0058	0.0057	0.0058	0.0058	0.0058	0.0057
b	0.0058	0.0092	0.0115	0.0138	0.0161	0.0184
b/a	1	1.61	1.98	2.38	2.78	3.23

**Table 2:** Decay times of the phosphorescence from the strontium aluminate crystals doped with various [Dy^3+^]/[Eu^2+^] ratios. Decay times were calculated by a curve fitting technique based on the three exponential components($\text{I}={\text{a}*\text{e}}^{-\frac{\text{t}}{\text{t}1}}+{\text{b}*\text{e}}^{-\frac{\text{t}}{\text{t}2}}+{\text{c}*\text{e}}^{-\frac{\text{t}}{\text{t}3}}$).

Sample #	#1	#2	#3	#4	#5	#6
t1[s]	330.2	357.3	370.7	371	326	337.8
t2[s]	19	20.22	21.61	21.17	19.93	20.45
t3[s]	0.1576	0.1419	0.03571	0.1112	0.2467	0.0154
a	187.1	198.8	208.3	220.7	190.7	166.6
b	846.8	870.4	877.3	893	835.5	716.9
c	0.925	0.9572	0.7922	0.3377	0.8452	0.2348

**Fig 2:**
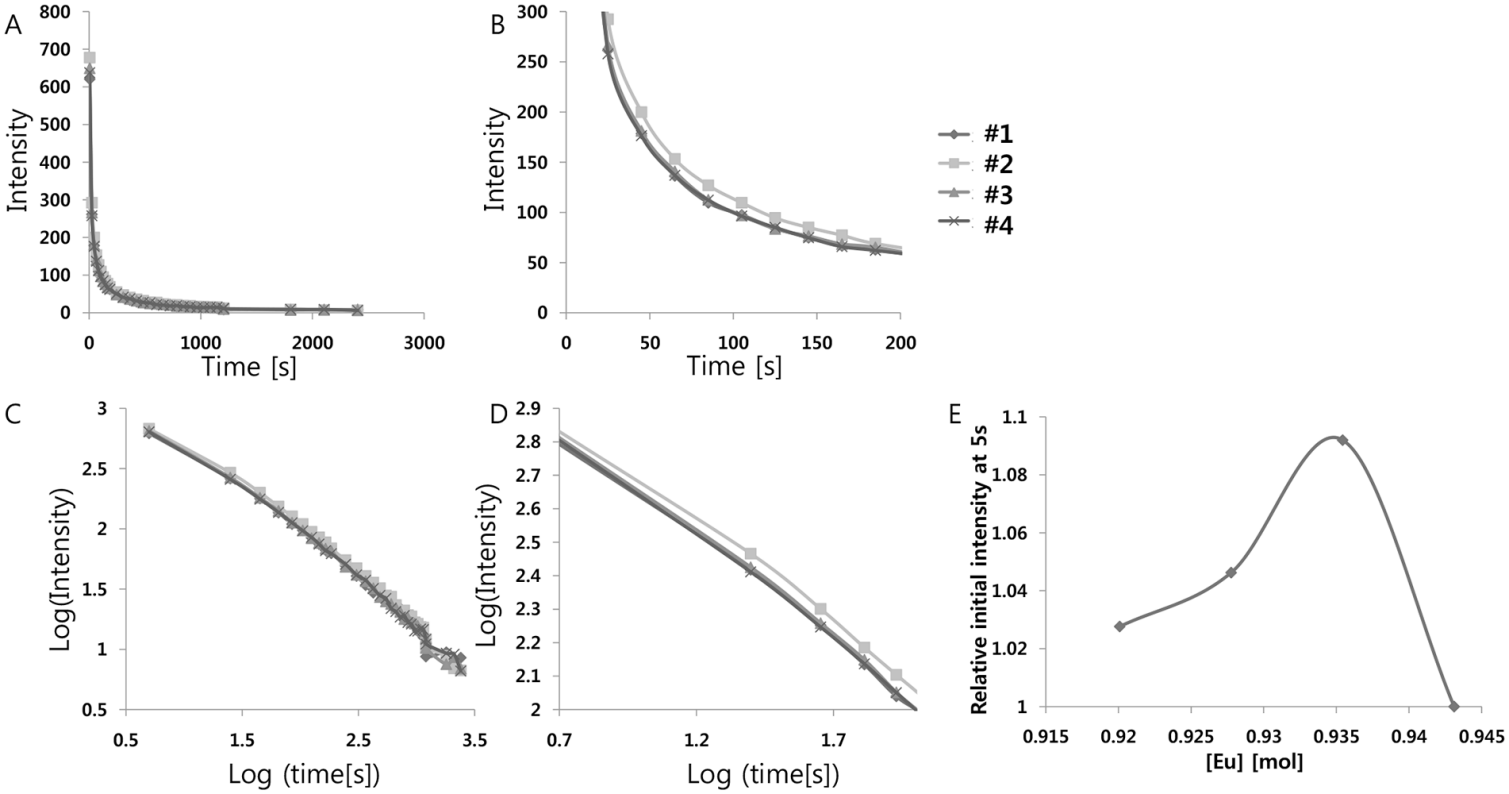
(A) Decay curves depending on Eu^2+^ concentration. (B) Magnified views of the graph in (A). (C) Decay curves in log scale depending on Eu^2+^ concentration. (D) Magnified views of the graph in (C). (E) Relative initial intensity measured at 5s (relative values where the value of control sample #1 is 1.0) depending on Eu^2+^ concentration.

Based on this result, we also tested different concentrations of Eu^2+^ at a constant Dy^3+^/Eu^2+^ ratio (~2.4) to find the optimal concentration of Eu^2+^. Eu^2+^ acts as an activator in strontium aluminate phosphors and affects the phosphorescence properties of the host, so that varying the amount of Eu^2+^ incorporated in the host lattice could significantly change the luminescence properties. The Eu^2+^ concentration was varied within the range from 0.920 to 0.942 mol (per mol of SrAl_2_O_4_:Eu^2+^,Dy^3+^), as shown in Table 3, to obtain bright phosphorescent phosphors; the result is shown in Fig 2 and Table 4. The observations illustrate that ~0.935 mol Eu^2+^ (per 1mol SrAl_2_O_4_:Eu^2+^,Dy^3+^) resulted in the brightest and longest emission. This change in luminescence properties with the amount of Eu^2+^ incorporated in the host lattice could be explained by significant changes in the local surroundings, such as bond length, bond angle, and point symmetry, around a substituted site.

**Table 3:** Various nominal activator(Eu^2+^) compositions of the strontium aluminate crystals.

SrAl_2_O_4_:Eu_c_,Dy_d_	#1	#2	#3	#4
c	0.0057	0.0069	0.0080	0.0092

**Table 4:** Decay times of the phosphorescence from the strontium aluminate crystals doped with various Eu^2+^ concentrations. Decay times were calculated by a curve fitting technique based on the three exponential components($\text{I}={\text{a}*\text{e}}^{-\frac{\text{t}}{\text{t}1}}+{\text{b}*\text{e}}^{-\frac{\text{t}}{\text{t}2}}+{\text{c}*\text{e}}^{-\frac{\text{t}}{\text{t}3}}$).

Sample #	1	2	3	4
t1[s]	343.1	339.9	334.6	336
t2[s]	18.14	18.45	17.48	21.4
t3[s]	0.1626	0.0119	0.2864	0.263
a	122.8	139.6	127.3	118.6
b	653.9	703.7	693.3	657.6
c	0.8782	0.934	0.5853	0.5285

### Doping with impurities

Since Eu^2+^ has a similar symmetry as Sr^2+^ due to their similar sizes (Sr^2+^: 132 pm, Eu^2+^: 131 pm) and charges, the host crystal structure is not changed significantly upon doping the strontium aluminate phosphor with Eu^2+^ [15]. It is known that the 5d→4f electronic transition of Eu^2+^ is sensitive to the symmetry of the coordination environment [13]. Therefore, further breaking of the symmetry could boost the luminescence by leading to less forbidden transitions. In order to break the symmetry of the host and create vacancies, we substituted Sr^2+^ with alkali metal or alkaline earth metal ions of various sizes, or substituted Al^3+^ with Si^4+^. These substitutions could lead to a strong local strain due to the differences in the ionic radii, thus enhancing the phosphorescence.

### Doping with impurities—Alkali metal doping

**Fig 3:**
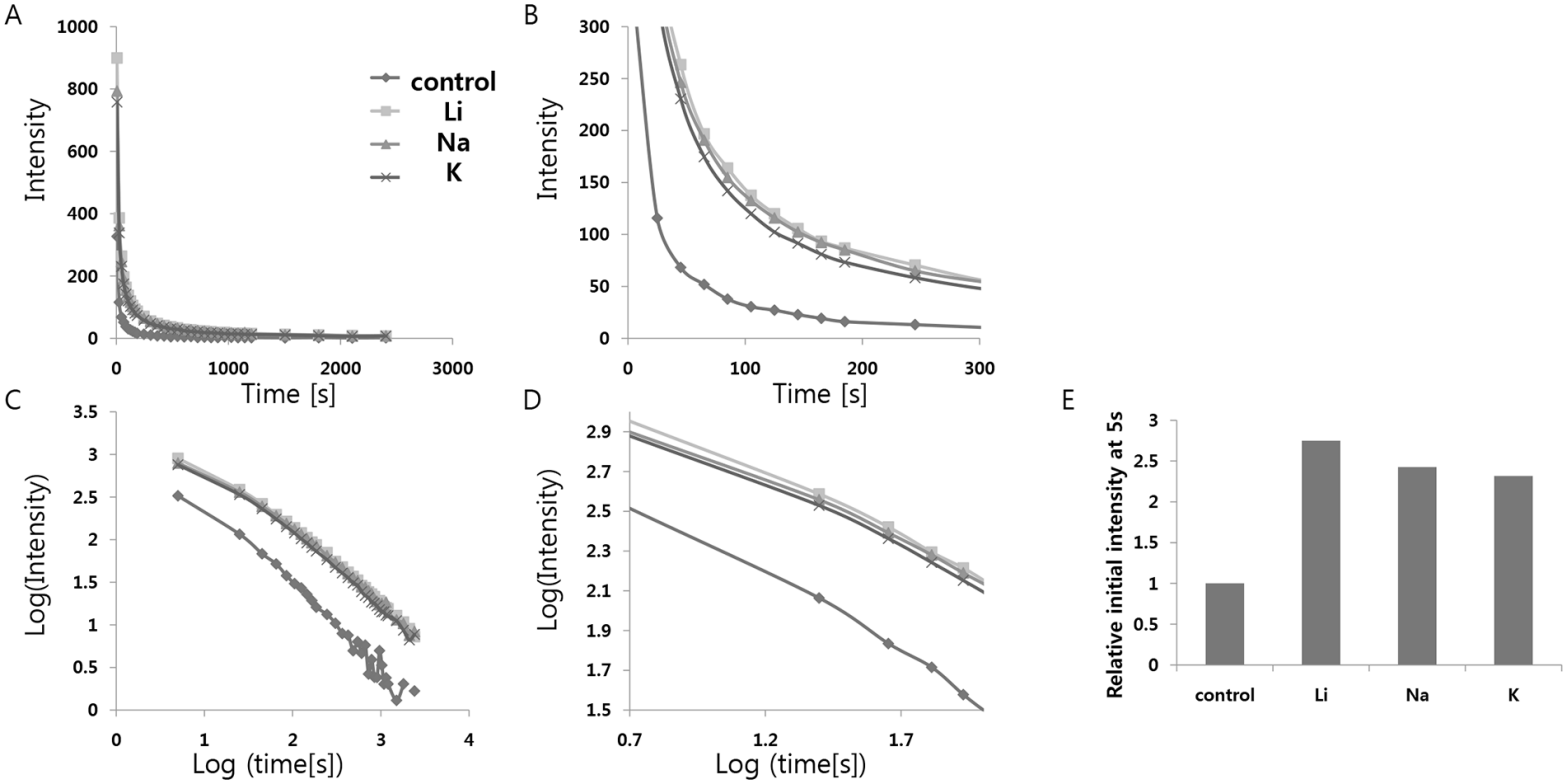
(A) Decay curves depending on alkali metal ion doping. (B) Magnified views of the graph in (A). (C) Decay curves in log scale depending on alkali metal ion doping. (D) Magnified views of the graph in (C). (E) Relative initial intensity measured at 5s (relative values where the value of control sample #1 is 1.0) depending on alkali metal ion doping.

First, we tried substitution of Sr^2+^ with alkali metals, which could decrease the number of cation vacancies, possibly inducing alternative relaxation paths for excitation energy. Since the excited 5d→4f transition of the Eu^2+^ ion is extremely sensitive to changes in the environment, we can also expect an additional increase in luminescence from the change in crystal structure symmetry caused by doping with alkali metals of different sizes, which could cause a corresponding shrinkage or expansion of the host structure. The distorted crystal structure may also facilitate the formation of traps, thus resulting in the improvement of initial afterglow characteristics. These two effects could boost the luminescence considerably, or induce little change in the luminescence if they cancel out each other. The ionic radii of the alkali metals increase smoothly from Li^+^ to K^+^(Sr^2+^: 132 pm, Li^+^: 90 pm, Na^+^: 116 pm, K^+^: 152 pm); Li^+^ and Na^+^ are smaller than Sr^2+^, while K^+^ is larger than Sr^2+^. All of the starting materials, SrCO_3_, Al_2_O_3_, Eu_2_O_3_, Dy_2_O_3_, SiO_2_, and M_2_CO_3_ (M = Li, Na, K), were weighed out and mixed homogeneously (Table 5). H_3_BO_3_ was added as a flux, and then, the dried powder mixtures were fired in the furnace. All of the afterglow measurements were performed subsequently; the curves in Fig 3 present the time dependences of the 520 nm emission. The phosphorescence spectrum due to Eu^2+^ ions, peaking at 520 nm, did not vary significantly with alkali metal doping, and the decay times of the SrAl_2_O_4_:Eu^2+^, Dy^3+^ doped with different alkali metal ions were almost similar(Table 6). However, the initial brightness of the phosphorescence after illumination was dramatically different. Alkali metal doping significantly increased the luminescence; especially, the smallest alkali metal, Li, showed the largest increase in luminescence, which supports our hypothesis that the distorted crystal structure may facilitate the formation of traps and enhance the afterglow characteristics.

**Table 5:** Nominal compositions of the strontium aluminate crystals doped with different alkali metals.

SrAl_2_O_4_:Eu_a_,Dy_b_	Control	Li	Na	K
mol	0	0.010	0.010	0.010

**Table 6:** Decay times of the phosphorescence from the strontium aluminate crystals doped with various alkali metals. Decay times were calculated by a curve fitting technique based on the three exponential components($\text{I}={\text{a}*\text{e}}^{-\frac{\text{t}}{\text{t}1}}+{\text{b}*\text{e}}^{-\frac{\text{t}}{\text{t}2}}+{\text{c}*\text{e}}^{-\frac{\text{t}}{\text{t}3}}$).

Sample	Control	Li	Na	K
t1[s]	340	295.4	315.2	291.9
**t2[s]**	20	18.24	19.65	19.32
**t3[s]**	0.8693	0.08113	0.3099	0.1111
**a**	118.6	188.1	173.1	161.7
**b**	751.2	933.8	797.4	770.1
**c**	0.2638	0.3685	0.4868	0.9049

**Fig 4:**
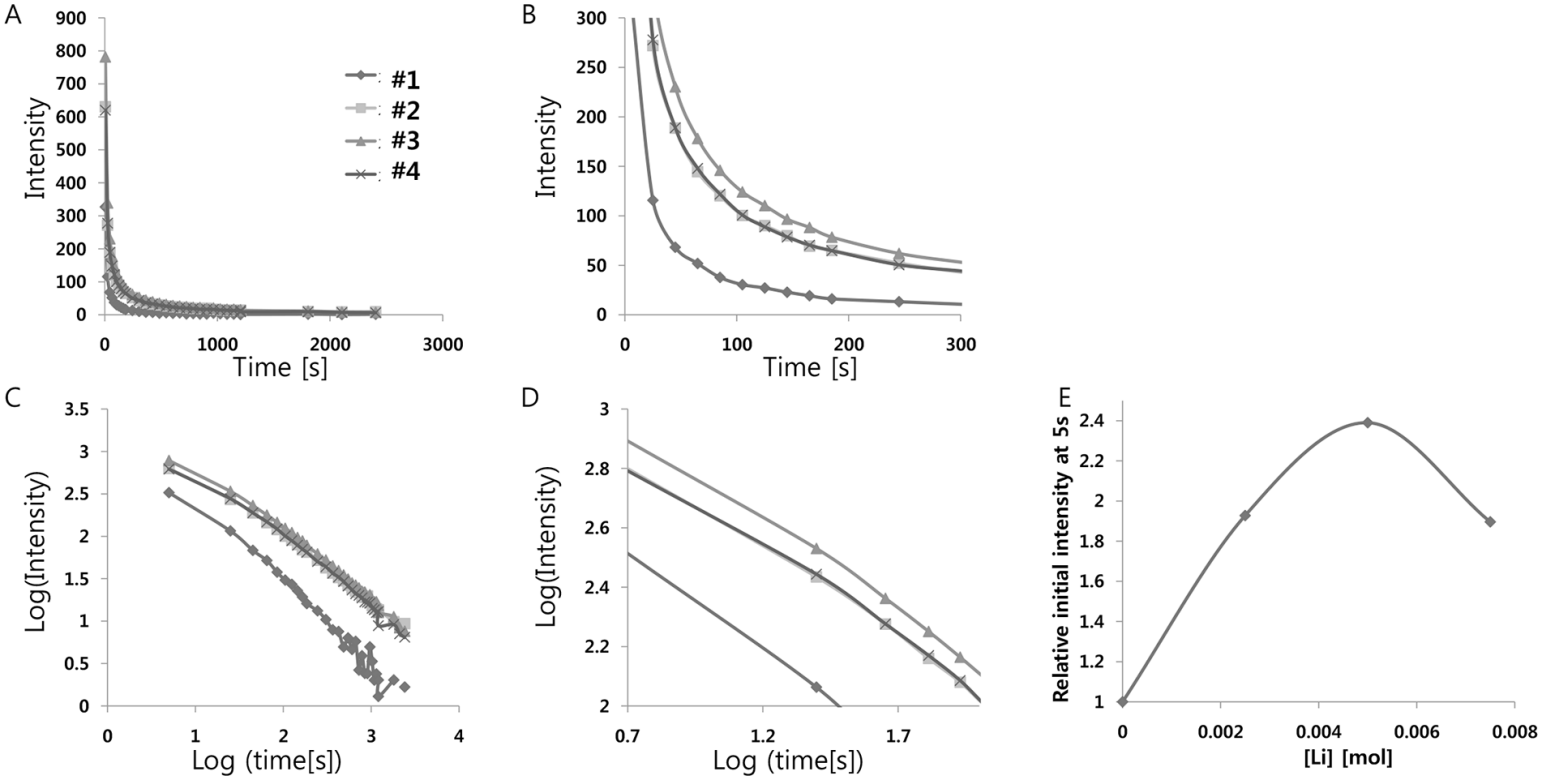
(A) Decay curves depending on Li^+^ concentration. (B) Magnified views of the graph in (A). (C) Decay curves in log scale depending on Li^+^ concentration. (D) Magnified views of the graph in (C). (E) Relative initial intensity measured at 5s (relative values where the value of control sample #1 is 1.0) depending on Li^+^ concentration.

We also tested various concentrations of Li_2_CO_3_ from 0.001 mol to 0.008 mol (per mol of SrAl_2_O_4_:Eu^2+^, Dy^3+^), in order to take full advantage of Li^+^ doping (Table 7). The position, shape, and width of the afterglow band did not change significantly with the concentration of Li^+^, indicating the same luminescent Eu^2+^ center. From the measurement (Fig 4 and Table 8), we found that the boost in phosphorescence intensity with Li^+^ doping ranges from 190% up to 239% of the initial value, depending on the concentration of Li^+^. The optimal concentration of Li_2_CO_3_ was 0.005 mol (per mol of SrAl_2_O_4_:Eu^2+^, Dy^3+^), which was enough to enhance the electronic transition of Eu^2+^ but not too high to disrupt the overall crystal structure.

**Table 7:** Nominal compositions of the strontium aluminate crystals doped with different Li^+^ concentrations.

SrAl_2_O_4_:Eu_a_,Dy_b_	#1	#2	#3	#4
mol	0	0.00250	0.00500	0.00750

**Table 8:** Decay times of the phosphorescence from the strontium aluminate crystals doped with various Li^+^ concentrations. Decay times were calculated by a curve fitting technique based on the three exponential components($\text{I}={\text{a}*\text{e}}^{-\frac{\text{t}}{\text{t}1}}+{\text{b}*\text{e}}^{-\frac{\text{t}}{\text{t}2}}+{\text{c}*\text{e}}^{-\frac{\text{t}}{\text{t}3}}$).

Sample	#1	#2	#3	#4
t1[s]	340	345.6	325.6	332.7
t2[s]	20	18.69	18.51	19.49
t3[s]	0.8693	0.2622	0.3205	0.02922
a	118.6	129.2	162.7	130.6
b	751.2	652.4	808.9	630.3
c	0.2638	0.2581	0.2217	0.08552

### Doping with impurities—Alkaline earth metal doping

**Fig 5:**
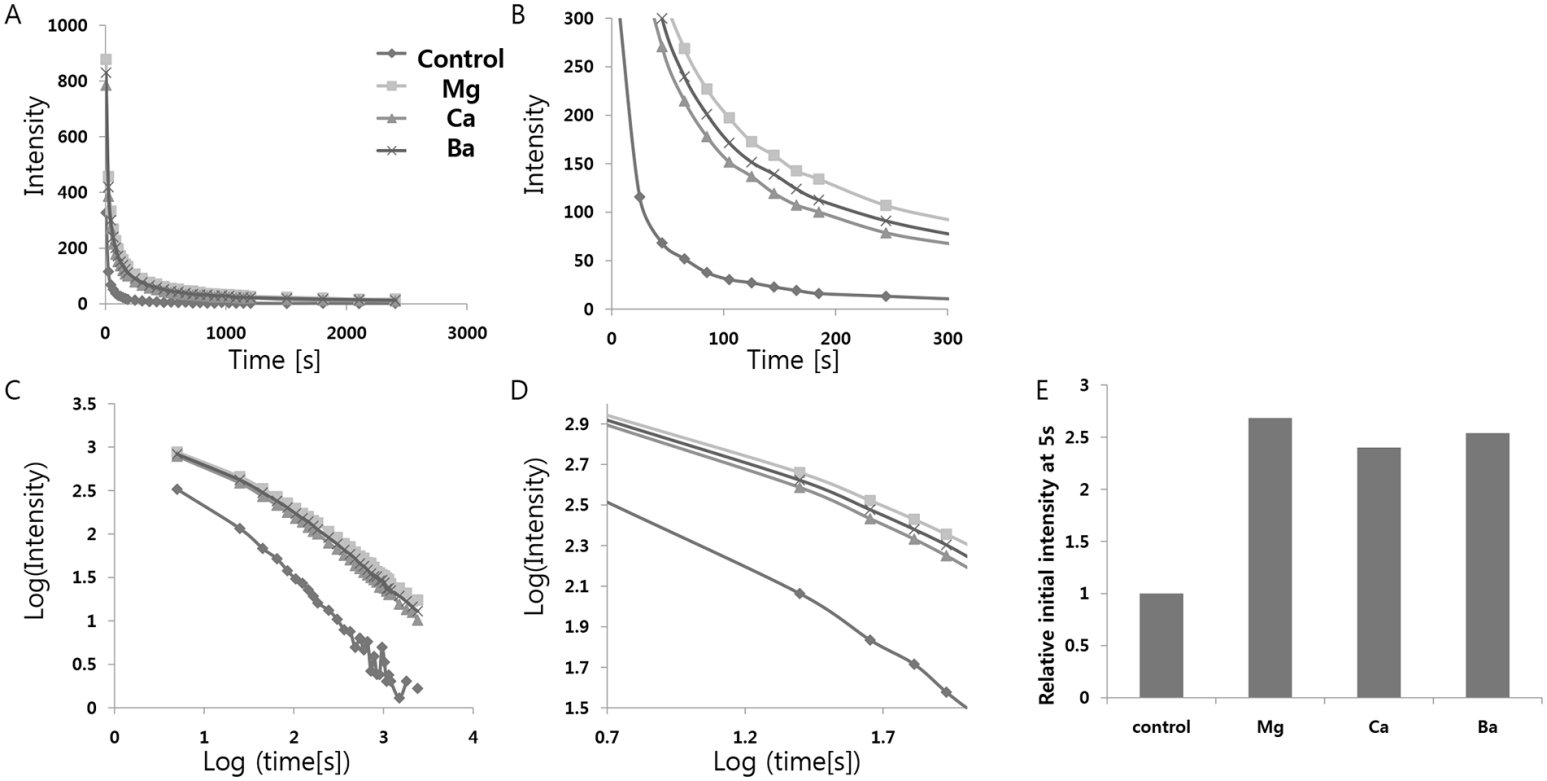
(A) Decay curves depending on alkali earth metal ion doping. (B) Magnified views of the graph in (A). (C) Decay curves in log scale depending on alkali earth metal ion doping. (D) Magnified views of the graph in (C). (E) Relative initial intensity measured at 5s (relative values where the value of control sample #1 is 1.0) depending on alkali earth metal ion doping.

Next, we tried to substitute Sr^2+^ in the strontium aluminate phosphor with alkaline earth metal ions of different sizes, to break the symmetry of the host crystal structure. The ionic radii of the alkaline earth metals increase smoothly from Mg^2+^ to Ba^2+^ in the body-centered cubic crystal structure (Mg^2+^: 86 pm, Ca^2+^:114 pm, Sr^2+^: 132 pm, Ba^2+^: 149 pm). Therefore, a break in symmetry of the host crystal structure is expected by expansion or shrinkage depending on the size of the ionic radii of a substitute, and hence may enhance the 5d→4f electronic transition of Eu^2+^. SrCO_3_, Al_2_O_3_, Eu_2_O_3_, Dy_2_O_3_, SiO_2_, H_3_BO_3_, and MCO_3_ (M = Mg, Ca, Ba) were weighed out and mixed homogeneously (Table 9), and then, the dried powder mixtures were fired in the furnace. All measurements of the decay curves of afterglow were performed subsequently, as shown in Fig 5. From the measurements (Fig 5 and Table 10), it is seen that the specimens exhibit broadband emission spectra peaking at 520 nm, and that the peak wavelengths of the phosphorescence spectra do not vary with the type of alkaline earth ions used in doping. It implies that the emission originates from the same Eu^2+^ center, and that the crystal field splitting and the center of gravity of Eu^2+^ are not influenced much by doping the SrAl_2_O_4_:Eu^2+^, Dy^3+^ crystals with the alkaline earth ions, but are likely fixed by the host network. However, as with alkaline metal doping, the initial intensities of the phosphorescence measured at 5s vary dramatically with the different alkaline earth metal ions used in doping. We observed an increase in luminescence of more than 2.5 times relative to the control sample with all three ions of different sizes. Among them, Mg^2+^ and Ba^2+^, whose sizes are significantly different from that of Sr^2+^, cause a greater increase in luminescence as compared to Ca^2+^. This suggests that the breaking of symmetry by doping with ions of different sizes could significantly enhance the 5d→4f electronic transition of Eu^2+^. The measurements with alkaline earth metals and alkali metals suggest that doping with ions of size ~90 pm (Li^+^:90 pm, Mg^2+^: 86 pm) results in the most significant enhancement in luminescence via appropriate changes in the crystal structure symmetry.

**Table 9:** Nominal compositions of the strontium aluminate crystals doped with different Alkaline Earth metal ions.

SrAl_2_O_4_:Eu_a_,Dy_b_	Control	Mg	Ca	Ba
mol	0	0.01	0.01	0.01

**Table 10:** Decay times of the phosphorescence from the strontium aluminate crystals doped with various alkaline earth metals. Decay times were calculated by a curve fitting technique based on the three exponential components($\text{I}={\text{a}*\text{e}}^{-\frac{\text{t}}{\text{t}1}}+{\text{b}*\text{e}}^{-\frac{\text{t}}{\text{t}2}}+{\text{c}*\text{e}}^{-\frac{\text{t}}{\text{t}3}}$).

	Control	Mg	Ca	Ba
t1[s]	340	341.9	337	331.6
t2[s]	20	18.48	17.52	16.95
t3[s]	0.8693	0.07818	0.004634	0.2602
a	118.6	139.1	126.8	127.2
b	751.2	703.8	693.4	684.6
c	0.2638	0.8258	0.4427	0.8687

**Fig 6:**
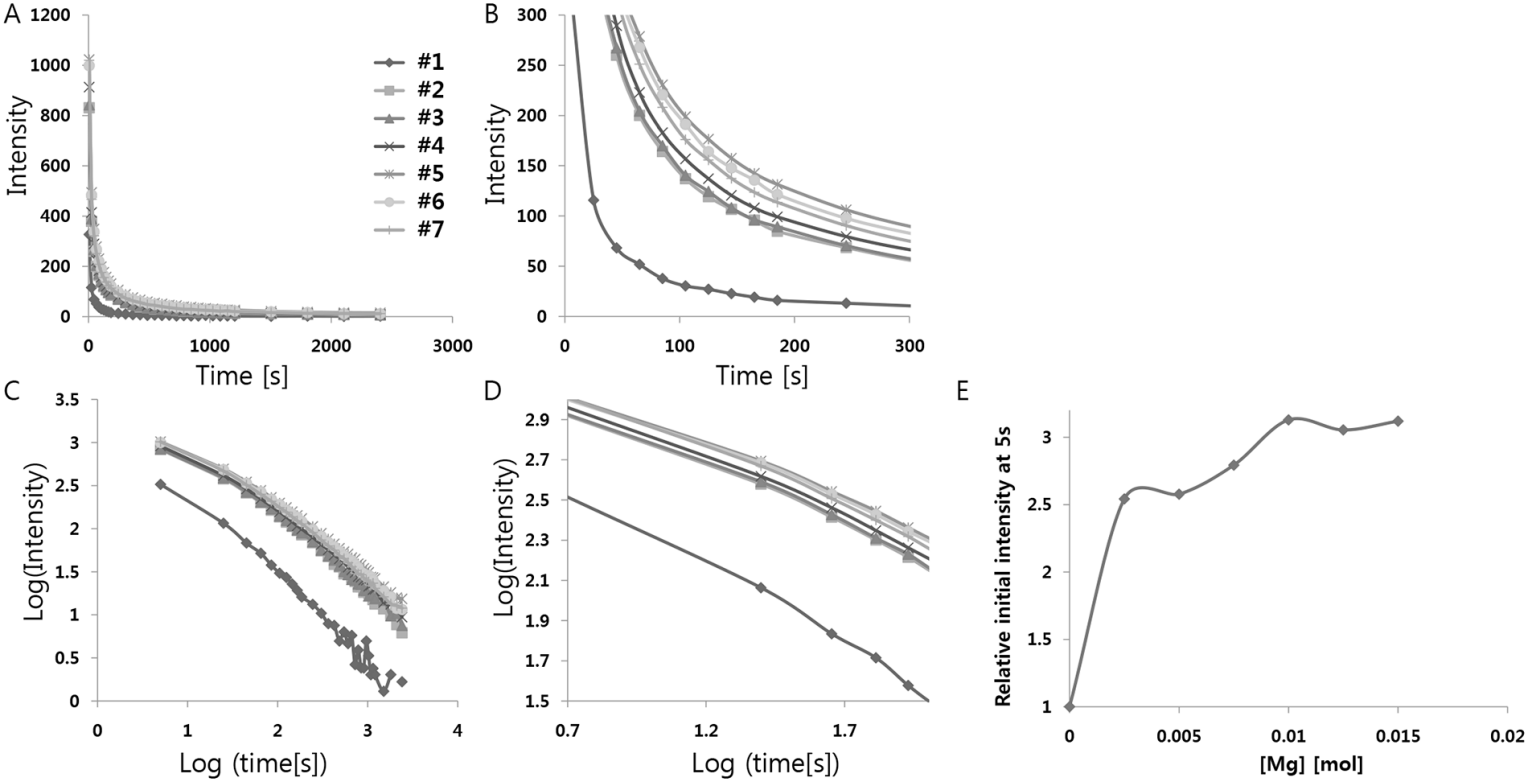
(A) Decay curves depending on Mg^2+^ concentration. (B) Magnified views of the graph in (A). (C) Decay curves in log scale depending on Mg^2+^ concentration. (D) Magnified views of the graph in (C). (E) Relative initial intensity measured at 5s (relative values where the value of control sample #1 is 1.0) depending on Mg^2+^ concentration.

Next, we synthesized different phosphors with various concentrations of MgCO_3_ to find the optimal concentration of Mg^2+^ (Table 11). In this case, we boosted the luminescence by doping with the optimized amount of Li^+^ as well, in order to further clarify the effect of breaking the centrosymmetry. Phosphors doped with various concentrations of MgCO_3_ (0 mol to 0.015 mol per mol of SrAl_2_O_4_:Eu^2+^, Dy^3+^) were tested; the result of the measurements is shown in Fig 6 and Table 12. All the afterglow bands from doping with different concentrations of Mg^2+^ had similar positions, shapes, and widths, indicating the same luminescent center. It is seen from this figure that the boost in phosphorescence intensity from Mg^2+^ doping, along with Li^+^ doping, ranges from 254% to 313% of the initial value, depending on the concentration of Mg^2+^. From the measurement, we found that the optimal concentration of Mg^2+^ is ~0.01 mol (per mol of SrAl_2_O_4_:Eu^2+^, Dy^3+^). A low concentration of Mg^2+^ (<0.01 mol) may not be enough to break the crystal structure symmetry, while a high concentration of Mg^2+^ (> 0.01 mol) could break the host crystal structure significantly and interrupt the electronic transition of Eu^2+^. These results can be explained by the hypothesis that increased diversity of doping to Eu^2+^ boosts the quantum yield, because the transitions become less forbidden in the mixed-doped complexes.

**Table 11:** Nominal compositions of the strontium aluminate crystals doped with different Mg^2+^ concentrations.

SrAl_2_O_4_:Eu_a_,Dy_b_	#1	#2	#3	#4	#5	#6	#7
Mol of Mg	0	0.0025	0.0053	0.0079	0.0105	0.0133	0.0159

**Table 12:** Decay times of the phosphorescence from the strontium aluminate crystals doped with various Mg^2+^ concentrations. Decay times were calculated by a curve fitting technique based on the three exponential components($\text{I}={\text{a}*\text{e}}^{-\frac{\text{t}}{\text{t}1}}+{\text{b}*\text{e}}^{-\frac{\text{t}}{\text{t}2}}+{\text{c}*\text{e}}^{-\frac{\text{t}}{\text{t}3}}$).

Sample	#1	#2	#3	#4	#5	#6	#7
t1[s]	340	293	300.1	338.9	381	357.8	325
t2[s]	20	19.5	19.96	19.84	21.24	21.42	19.57
t3[s]	0.8693	0.4882	0.663	0.4318	0.2931	0.1056	0.1078
a	118.6	186.7	188.7	197.8	239.2	231.4	229
b	751.2	830.9	837.7	916.3	985.6	963.5	1018
c	0.2638	0.1335	0.1839	0.1634	0.9577	0.9686	0.7302

### Doping with impurities—Si^4+^ doping

**Fig 7:**
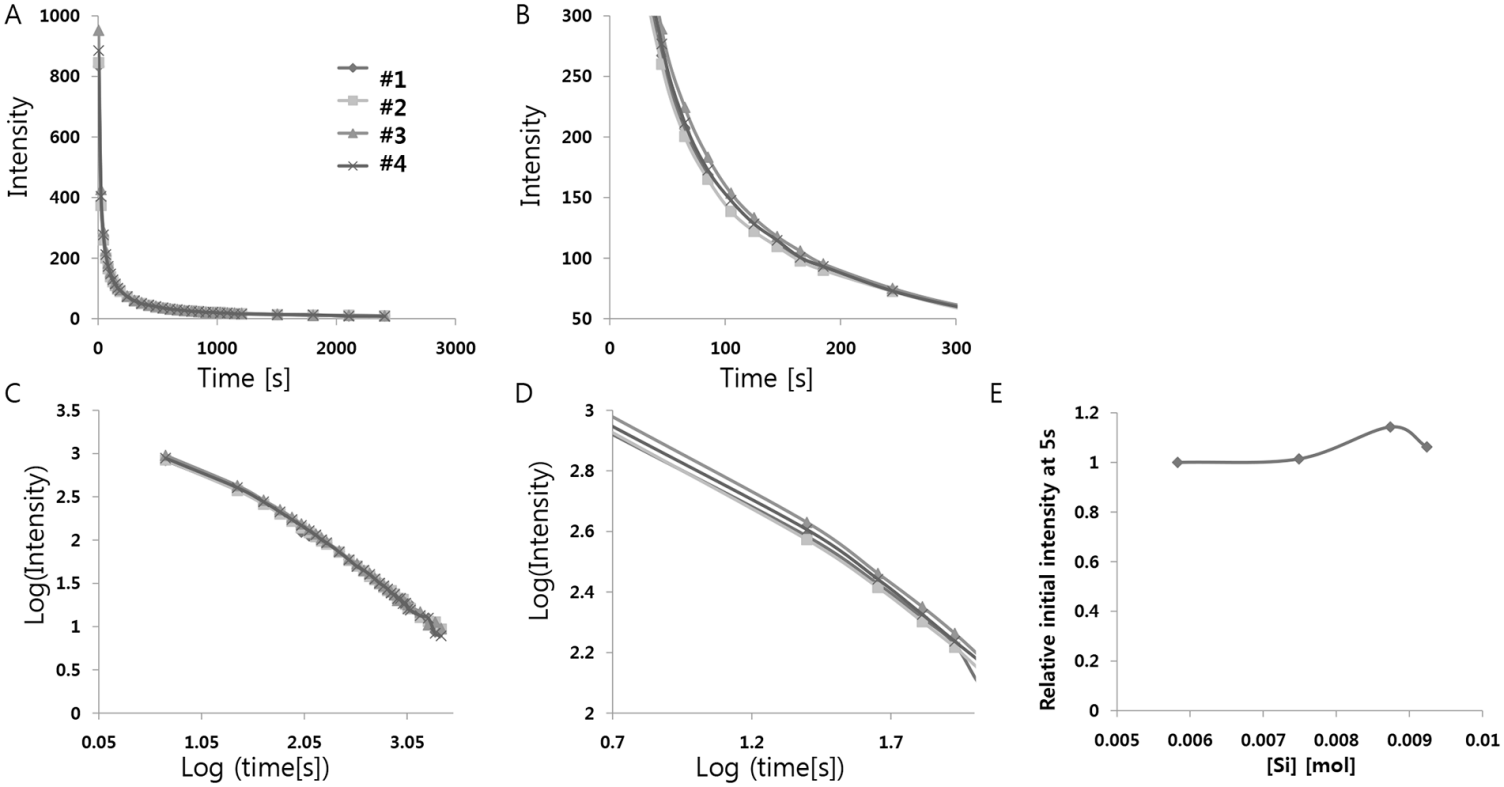
(A) Decay curves depending on Si^4+^ concentration. (B) Magnified views of the graph in (A). (C) Decay curves in log scale depending on Si^4+^ concentration. (D) Magnified views of the graph in (C). (E) Relative initial intensity measured at 5s (relative values where the value of control sample #1 is 1.0) depending on Si^4+^ concentration.

Next, we performed Si^4+^ doping experiments in order to substitute Al^3+^ with Si^4+^and create a cation vacancy, which could cause expansion of the host structure. Moreover, cation vacancies are expected to act as hole traps and enhance the phosphorescence if the depth is optimal to show long phosphorescence at room temperature; this is because the co-activator Dy^3+^ greatly enhances the duration and intensity of persistent luminescence by creating highly dense hole trapping levels. We can expect both the effects of breaking the symmetry due to the different ion sizes and creation of a vacancy from the different charges of the ions. The size of Si^4+^ is ~40 pm in tetrahedral coordination, which is smaller than the size of Al^3+^ (53 pm), suggesting shrinkage of the crystal structure upon the substitution of Si^4+^. In addition, creation of a cation vacancy by the substitution of Si^4+^ can cause expansion of the host structure. These two different effects would boost the luminescence if they are synergistic, or induce very little change in the luminescence if they cancel out each other. We added various concentrations of SiO_4_ (0.005–0.02M per 1mol SrAl_2_O_4_:Eu^2+^, Dy^3+^) as shown in Table 13. The decay curve of afterglow was measured at room temperature after irradiation with 365 nm light for 5 min (Fig 7 and Table 14). A slight increase (~15%) in luminescence was seen upon SiO_2_ doping, and the optimal SiO_2_ concentration for the strongest luminescence was 0.00875 mol (per mol of SrAl_2_O_4_:Eu^2+^, Dy^3+^). These small changes in luminescence are most likely due to the fact that the shrinking effect caused by the smaller size of Si^4+^ cancels out the expansion effect resulting from a cation vacancy. Another possibility is that the substitution of Sr^2+^ could be more effective than the substitution of Al^3+^ for breaking the symmetry of the crystal structure.

**Table 13:** Nominal compositions of the strontium aluminate crystals doped with different Si^4+^ concentrations.

SrAl_2_O_4_:Eu_a_,Dy_b_	#1	#2	#3	#4
Mol of Si	0.00583	0.00749	0.00874	0.00924

**Table 14:** Decay times of the phosphorescence from the strontium aluminate crystals doped with various Si^4+^ concentrations. Decay times were calculated by a curve fitting technique based on the three exponential components($\text{I}={\text{a}*\text{e}}^{-\frac{\text{t}}{\text{t}1}}+{\text{b}*\text{e}}^{-\frac{\text{t}}{\text{t}2}}+{\text{c}*\text{e}}^{-\frac{\text{t}}{\text{t}3}}$).

Sample	#1	#2	#3	#4
t1[s]	354.3	318.8	292.9	309.8
t2[s]	21.16	18.88	19.05	19.74
t3[s]	0.3059	0.06359	0.2648	0.4344
a	169.3	184.5	209.1	193.4
b	835.2	858.2	964	888.5
c	3.429	0.2187	0.5822	0.2503

## Discussion

Here, we report a systematic investigation of the Eu-doped alkaline earth aluminate SrAl_2_O_4_:Eu^2+^, Dy^3+^ crystals grown with various compositions, with the aim of developing bright and persistent phosphors. From the composition studies on the activator Eu^2+^ and the co-activator Dy^3+^, we found that the Eu^2+^- and Dy^3+^-doped strontium aluminates with a Dy^3+^/Eu^2+^ ratio of ~2.4 showed the strongest persistent luminescence (11% enhancement), and that ~0.935 mol Eu^2+^ (per mol of SrAl_2_O_4_:Eu^2+^, Dy^3+^) resulted in the brightest and longest emission (9% enhancement). The persistent luminescence intensity can be enhanced further with the addition of alkali metal or alkaline earth metal ions. In particular, doping with 0.005 mol Li^+^ alone (per mol of SrAl_2_O_4_:Eu^2+^, Dy^3+^) boosts the phosphorescence intensity to 239% of the initial value as compared to the non-doped crystal. Doping with 0.01 mol Mg^2+^ and 0.005 mol Li^+^ (per 1mol SrAl_2_O_4_:Eu^2+^, Dy^3+^) boosts the phosphorescence intensity to 313% of the initial value. Meanwhile, Si^4+^ doping affords a slight increase (up to 15%) in luminescence, and the optimal SiO_2_ concentration for the brightest luminescence is 0.00875 mol (per mol of SrAl_2_O_4_:Eu^2+^, Dy^3+^). Therefore, we could improve the initial afterglow characteristics of SrAl_2_O_4_:Eu^2+^, Dy^3+^ crystals under the excitation condition of low illumination, so that the afterglow characteristics are superior to those of conventional phosphorescent phosphors. Further studies using different experimental and spectroscopic techniques are needed to establish in detail the effects of composition on the phosphorescence of the Eu-doped alkaline earth aluminate SrAl_2_O_4_:Eu^2+^, Dy^3+^ crystals. We anticipate this protocol to open up an unexpectedly large field of applications for the use of these aluminates, for example, in the areas of safety and energy saving.

## Methods

The starting materials were high-purity SrCO_3_, Al_2_O_3_, Eu_2_O_3_ (Rhône-Poulenc, 99.99%), Dy_2_O_3_, MCO_3_ (M = Ca, Sr, Ba; Merck, > 99.0%), and SiO_2_ (Aerosil OX 50, Degussa). Small quantities of H_3_BO_3_ or B_2_O_3_ (0.1–0.3M) were added as a flux. The starting materials were weighed out in various amounts, mixed homogeneously, and ground in an agate mortar. Then, the dried powder mixtures were fired in molybdenum crucibles at ~1300°C for 3–5 h, under a weak reductive atmosphere of flowing N_2_-H_2_ (3%) gas, in horizontal tube furnaces. After a high-temperature solid-state reaction, the synthesized samples were cooled to room temperature in the furnace, and were ground again in an agate mortar. For the afterglow measurements, the samples were irradiated with 365 nm light for 5 min, and the emission spectra of the phosphors were recorded by a Hitachi 850 fluorescence spectrophotometer, from 300 to 950 nm. The decay curves of afterglow were measured with an ST-86LA brightness meter. All measurements were carried out at room temperature.
